# Study of mental health perceptions among Central African refugee populations and host communities in the East Cameroon region

**DOI:** 10.1192/j.eurpsy.2024.365

**Published:** 2024-08-27

**Authors:** E. Dozio, A. Jaccard

**Affiliations:** ^1^Action contre la Faim, Paris, France; ^2^Action contre la Faim, Yaoundé, Cameroon

## Abstract

**Introduction:**

Cameroon’s eastern region faces numerous security challenges linked to successive crises in the Central African Republic, particularly with the massive influx of refugees especially since 2013. Official UNHCR figures speak of 349,409 Central African refugees present on Cameroonian soil. These are both refugees already well established in their host communities, and new arrivals. Since the post-electoral crisis in CAR at the end of 2020, the situation has gradually stabilized in the Kadey department, but remains volatile due to daily insecurity in the northern regions of the Central African Republic.

**Objectives:**

With a view to meeting the mental health and psychosocial support needs of the region’s population and better integrating refugees into their host communities, it was necessary to obtain a more exhaustive picture of the population’s perception of mental health, to understand the mechanisms of psychosocial support at community level and any differences between refugees and the indigenous population.

**Methods:**

A mixed methodology with quantitative and qualitative data was chosen for a more detailed analysis. The survey was carried out in two communes in the Kadey department: Kentzou and Kette. The sample was disaggregated to take account of the socio-demographic characteristics and to enable to make comparisons between the situations of host communities, living in refugee sites and outside sites. For quantitative data, 205 the individuals responded to a questionnaire. 12 individual interviews and 12 Focus Group Discussions (involving 60 participants ) guided by semi-structured questions were used to collect qualitative data from key members of the community.

**Results:**

Analysis of quantitative and qualitative data has confirmed the successful integration of Central African refugees into the host community. Nevertheless, there is a difference between these two groups in terms of their perception and understanding of the definition of mental health, mental health disorders and treatment options. Thus, there was a clear difference in training and awareness needs between the host and refugee communities.

**Conclusions:**

Based on the qualitative and quantitative results of our assessment, a number of recommendations have been drawn up. It would be interesting to find a balance in the definition, attribution of causes and treatment of mental disorders between the traditional, mystical and cultural vision and the psychological and psychiatric one. It would be important to encourage refugee communities to return to some of their traditional practices, and to allow several visions of the human being to coexist between a traditional and a more medical vision.

To address the lack of resources for mental health care in the Kadey area, more training in mental health and psychosocial support is needed for health and social workers in the area.

**Disclosure of Interest:**

None Declared

